# Prognostic value of CD47 overexpression measured by flow cytometry in acute myeloid leukemia

**DOI:** 10.1007/s00277-025-06401-2

**Published:** 2025-05-15

**Authors:** Vincenzo Sammartano, Anna Sicuranza, Paola Pacelli, Elena Bestoso, Adele Santoni, Corrado Zuanelli Brambilla, Marzia Defina, Alessandra Cartocci, Donatella Raspadori, Monica Bocchia

**Affiliations:** 1 Hematology Unit, Department of Medical Science, Surgery and Neurosciences, AOUS Policlinico Le Scotte, University of Siena, Siena, Italy; 2 Department of Clinical Medicine and Surgery, Hematology Section, University of Naples Federico II, Naples, Italy; 3 Department of Medical Science, Surgery and Neurosciences, University of Siena, Siena, Italy

**Keywords:** CD47, Acute myeloid leukemia, Tumor microenvironment

## Abstract

The glycoprotein CD47 is an innate immune checkpoint ubiquitously expressed on all healthy cells to prevent themselves from phagocytosis. CD47 binds to its receptor SIRPα on macrophages, thus producing a signal transduction cascade which inhibits phagocytosis. CD47 is overexpressed on various solid and hematologic malignancies in order to escape the immune system. High expression of CD47 in patients with AML has been associated with poor prognosis, however, there is no standard technique to assess CD47 expression on AML blasts in clinical practice and the real prognostic value of CD47 overexpression varies among studies in the current literature. In this study, CD47 expression was evaluated by flow cytometry on AML blasts from bone marrow samples at diagnosis and reported in terms of median fluorescence intensity (MFI). Flow cytometry analysis demonstrated the expression of CD47 in all AML patients with a median MFI on leukemic blasts of 16.8 (range 2–693.63). CD47 levels on AML blasts correlated with WBC count (rs 0.403, *p* = 0.016), BM blasts percentage (rs 0.494, *p* = 0.003), PB blasts percentage (rs 0.482, *p* = 0.003) and LDH levels (rs 0.382, *p* = 0.028) and higher expression of CD47 was associated with reduced survival with a hazard ratio of 1.04 (CI: 1.01–1.08, *p* = 0.047). Further studies with larger sample sizes are necessary to better define the real prognostic value of CD47 overexpression in the complexity of AML tumor microenvironment and, possibly, to identify a subgroup of patients who could derive maximum benefit from emerging CD47-SIRPα blocking therapies.

## Introduction

Acute myeloid leukemia (AML) is a heterogenous blood cell cancer characterized by uncontrolled proliferation of clonal hematopoietic cells [1]. AML is the most common acute leukemia in adults with a median age at diagnosis of 68 years [2]. The estimated 5-year overall survival (OS) is 30%, however, it is less than 10% in patients older than age 60 [3].

The cell surface glycoprotein CD47 (also called integrin-associated protein) is a critical “don’t eat me” signal to the innate immune system, which inhibits phagocytosis by binding the signal regulatory protein alpha (SIRPα), an inhibitory immunoreceptor on macrophages [4].

CD47 is ubiquitously expressed on all normal cells to prevent themselves from phagocytosis, however, it was found to be also overexpressed on various solid and hematologic tumor cells in order to escape the immune system [5]. This mechanism of immune evasion is similar to the PD1-PD-L1 pathway, where PD-L1 is overexpressed in cancer cells and binds to PD1 expressed on T cells resulting in T cells exhaustion and tumor evasion from adaptive immune system [6].

Hematological malignancies express high levels of CD47 as interactions between CD47 on cancer cells and SIRPα on macrophages inhibit phagocytosis resulting, thus, in tumor immune evasion [7]. High expression of CD47 in patients with AML has been associated with poor prognosis and disease relapse [8, 9]. However, there is no standard technique to assess CD47 expression on AML blasts in clinical practice and the real prognostic value of CD47 overexpression varies among studies [10–12].

Since outcomes of refractory and relapsed AML patients are consistently disappointing, novel therapeutic approaches are warranted and CD47-SIRPα blockade has emerged as a possible novel therapeutic strategy. Indeed, anti-CD47 antibodies or SIRPα-Fc fusion proteins inhibit the “don’t eat me” signal by blocking the CD47- SIRPα axis and unmask the “eat me” (pro-phagocytic) signals expressed on cancer cells, thus promoting macrophage phagocytosis. Normal cells usually lack the expression of pro-phagocytic signals, consequently they are spared by CD47 blocking agents [13]. Magrolimab, a first-in-class anti-CD47 antibody, has initially shown promising activity results when combined with azacitidine in AML patients, however, phase 3 trials were recently interrupted for futility, non-efficacy, and increased mortality. Despite that, several other CD47/SIRPα targeting agents are being studied in pre-clinical and clinical trials, showing the high level of interest for targeting this pathway. Different approaches to targeting the CD47 pathway are being investigated, which may lead to different safety and efficacy profiles in the clinical setting. Indeed, CD47 blockade by itself is not sufficient to activate macrophage phagocytosis, which also needs a pro-phagocytic signal. For this reason, many novel CD47-SIRPα targeting agents under investigation are developed to not only block CD47, but also to trigger the activating Fc gamma receptor (FcγR) on macrophages in order to provide a stronger phagocytic signal to macrophages [14].

Considering the potential of CD47 as an independent prognostic marker in AML, the aim of this study was to investigate the correlation between CD47 expression on bone marrow blasts and clinical outcome in AML patients.

## Methods

Newly diagnosed AML patients referred to our center (Hematology Unit, Azienda Ospedaliera Universitaria Senese, Siena, Italy) from November 2021 to June 2024 were included in this study. Patients with the acute promyelocytic leukemia (APL) were excluded from the study group. There were no exclusion criteria regarding medical fitness, ECOG performance status or comorbidities of any degree. This study received approval from regulatory authorities and was conducted according to the Declaration of Helsinki, the International Conference on Harmonization, and the Guidelines for Good Clinical Practice. All patients provided written informed consent prior to entering the study. AML diagnosis was made according to either the International Consensus Classification (ICC) or World Health Organization 5th edition (WHO5) criteria [15, 16]. All cases received prompt cytogenetic and molecular investigations to define risk stratification and, consequently, the treatment choice and response criteria according to the 2022 ELN risk classification [17].

MRD was assessed by multiparameter flow cytometry (MFC-MRD) in case of leukemia-associated immunophenotype (LAIP) or “different from normal” immunophenotype and by quantitative polymerase chain reaction (qPCR) for molecular MRD (Mol-MRD) in cases with NPM1 mutation, CBFB::MYH11, or RUNX1::RUNX1T1.

CD47 expression was evaluated by flow cytometry on AML blasts from bone marrow (BM) samples performed at diagnosis. Flow cytometry analysis was performed by using an antigen panel that comprises PerCP Cy5.5–conjugated CD34, V500-conjugated CD45 and PE-conjugated CD47. A negative control tube has been used with the same combination of PerCP Cy5.5–conjugated CD34 and V500-conjugated CD45, mixed with a PE-conjugated Isotype Control. Cells were detected by using a multicolor BD FACSLyric flow cytometry instrument and BD FACSuite software (BD Biosciences) with a protocol that implied adjustments of FACS parameters, using the CS&T System (BD Biosciences), to keep the instrument performance constant by correcting wear of lasers and fluidic instability.

**Fig. 1:**
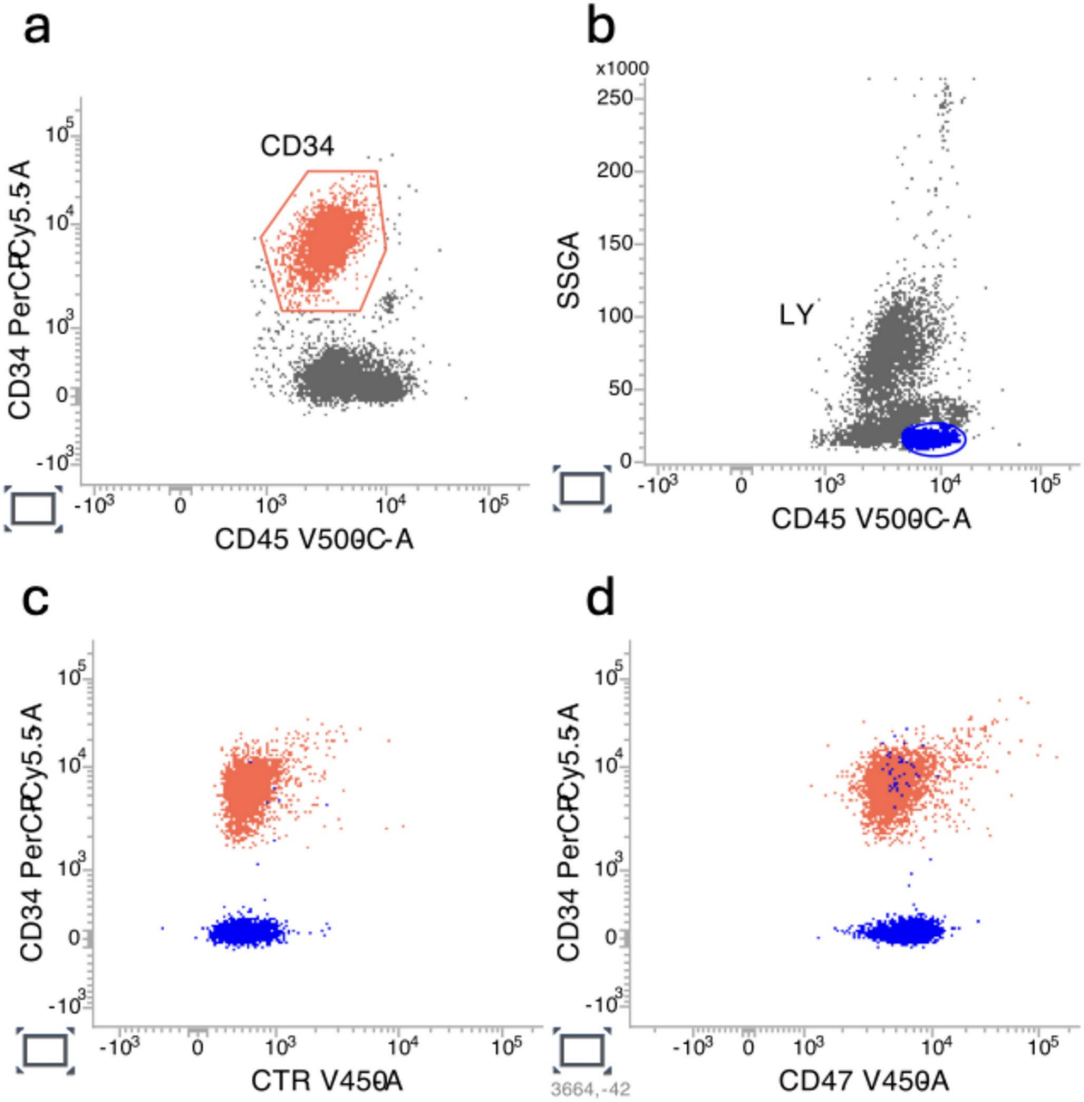
Identification by flow cytometry of the CD47 expression on AML blasts and the residual normal lymphocytic population

In all cases, CD47 expression was expressed in terms of median fluorescence intensity (MFI) and was compared with the residual normal lymphocytic population. PD-L1 on AML blasts was also evaluated by flow cytometry. Figure 1 represents a case of CD47 expression by flow cytometry on AML blasts and the residual normal lymphocytic population.

The prognostic impact of CD47 expression was statistically determined. CD47 expression on AML blasts was correlated with clinical features and outcomes; correlations of variables were computed with Spearman’s rank correlation coefficient. ROC analysis was performed to evaluate the predictive ability of CD47. Survival analysis was performed using the Cox regression and the Kaplan-Meier methods. OS was defined as time from diagnosis to death, while progression-free survival (PFS) was defined as time between initiation of treatment and progression or death. *P* < 0.05 was considered statistically significant. Statistical analyses were performed using R version 4.3.1.

## Results

During the study core, 35 patients were included. The median age was 70 years (range, 20–87), 19 males and 16 females. A total of 22/35 (62.8%) patients presented de novo AML, while 13/35 (37.2%) had secondary AML (11 progressing from myelodysplastic syndromes (MDS) or myeloproliferative neoplasms (MPN) and 2 therapy-related). At baseline, the median blast percentage was 60% (range 15–100) on BM aspirates and 12% (range 0–100) on peripheral blood (PB). Median white blood cell (WBC) count at baseline was 11.27 × 10^9^/L (range 0.7–193.62), median hemoglobin (Hb) level was 9.5 g/dL (range 6.8–15.3), median PLT count was 47 × 10^9^/L (range 8–310) and median LDH level (normal range 140–250 U/L) was 454 U/L (range 199–2544). According to the 2022 ELN risk classification, 7/35 (20%) patients presented favorable risk, 14/35 (40%) intermediate risk, and 14/35 (40%) adverse risk.

A total of 21/35 (60%) patients were considered fit for intensive therapy, while 14/35 (40%) were considered unfit. Among the fit patients, 8/21 (38.1%) patients were treated with “7 + 3” regimen (of which 4 plus midostaurin and 3 plus gemtuzumab ozogamicin), 7/21 (33.3%) with FLAI (fludarabine, cytarabine, idarubicin) regimen, and 6/21 (28.6%) with CPX-351. Among the unfit patients, 7/14 (50%) received azacitidine plus venetoclax, 5/14 (35.7%) decitabine plus venetoclax, and 2/14 (14.3%) low dose cytarabine (LDAC). Allogeneic HCT was performed in 9/35 (25.7%) patients. Table 1 summarizes baseline patient and disease characteristics and type of induction therapy.

**Table 1:** Patient and disease characteristics at baseline and type of induction therapy

Characteristcs	Patients (*n*=35)
Age at diagnosis, median	70 years (range 20–87)
De novo AML	22/35 (62.8%)
Secondary AML	13/35 (37.2%)
BM blasts, median	60% (range 15–100)
PB blasts, median	12% (range 0–100)
WBC, median	11.27 × 10^9^/L (range 0.7–193.62)
Hb, median	9.5 g/dL (range 6.8–15.3)
PLT, median	47 × 10^9^/L (range 8–310
LDH, median	454 U/L (range 199–2544)
2022 ELN risk classification	
• favorable • intermediate • adverse	• 7/35 (20%) • 14/35 (40%) • 14/35 (40%)
Medical fitness for intensive therapy	
• fit • unfit	• 21/35 (60%) • 14/35 (40%)
Intensive induction therapy • 7 + 3 • FLAI • CPX-351	21/35 (60%) • 8/21 (38.1%) • 7/21 (33.3%) • 6/21 (28.6%)
Non-intensive induction therapy • Azacitidine plus venetoclax • Decitabine plus venetoclax • LDAC	14/35 (40%) • 7/14 (50%) • 5/14 (35.7%) • 2/14 (14.3%)

a. AML blasts population is identified by the expression of CD34

b. Normal lymphocytic population is identified by the expression of CD45 at high intensity

c. Negative control expression in both populations

d. CD47 expression in both populations

At response evaluation after induction therapy, 17/35 (48.6%) patients achieved CR, of which 8/17 (47%) obtained MRD negativity status, while 18/35 (51.4%) did not respond and had refractory disease. Among fit patients treated with intensive chemotherapy, CR was achieved by 13/21 (61,9%) patients (7/13 (53.8%) MRD negative). Among unfit patients treated with hypomethylating agent (HMA) with or without venetoclax or LDAC, CR was achieved by 4/14 (28.6%) patients (1/4 (25%) MRD negative). Relapse occurred in 8/17 (47%) patients. At the time of writing, no patient has been lost to follow-up. Overall, 20/35 (57.1%) were alive at the end of data collection, while 15/35 (42.9%) patients died and mostly of deaths were AML related or due to concomitant infection. Median OS was 23 months (95% CI, 7–23) and median PFS was 2 months (95% CI, 1–6). Among fit patients, median OS was not reached and median PFS was 4 months (95% CI, 1–16). Among unfit patients, conversely, median OS was 7 months (95% CI, 3–13) and median PFS was 1 months (95% CI, 1–6).

Flow cytometry analysis demonstrated the expression of CD47 in all AML patients with a median MFI on leukemic blasts of 16.8 (range 2–693.63). We did not observe different CD47 expression between de novo and secondary AML (median MFI 17.9 vs. 16.8, respectively). In 5/35 (14.3%) patients the expression of CD47 in leukemic blasts was higher than in the residual normal lymphocytic population. PD-L1 expression on AML blasts was evaluated by flow cytometry in 31/35 (88.6%) cases and in 9/31 (29%) cases PD-L1 was not expressed. PD-L1 was expressed on leukemic blasts in 22/31 (71%) cases with a median MFI of 40.22.

No significant differences were observed in patients with higher expression of CD47 on AML blasts than in the control lymphocytic population. However, when we considered CD47 expression as a continuous variable, Spearman’s rank correlation analysis demonstrated correlations with WBC count (r_s_ 0.403, *p* = 0.016), BM blasts percentage (r_s_ 0.494, *p* = 0.003), PB blasts percentage (r_s_ 0.482, *p* = 0.003) and LDH level (r_s_ 0.382, *p* = 0.028). CD47 expression did not correlate with the 2022 ELN risk classification and with genetic abnormalities. No therapeutic approach was associated to better response rates or improved survival in patients with higher expression of CD47. No statistical correlations between CD47 and PD-L1 expression were found. ROC analysis showed no high predictive ability of CD47 expression for death (AUC 0.57, 95% CI: 0.38–0.77), relapse (AUC 0.64, 95% CI: 0.35–0.92), or response to treatment (AUC 0.67, 95% CI: 0.48–0.86). For this reason, it was not possible to determine a cut-off level to discriminate AML cases with low or high CD47 expression. However, Cox regression analysis determined that higher expression of CD47, considered as a continuous variable, was associated with reduced OS with a hazard ratio (HR) of 1.04 (CI: 1.01–1.08, *p* = 0.047), yet it was not associated with PFS (HR 1, CI 0.97–1.03, *p* = 0.969). Additionally, in univariate analysis, significant associations were observed for ECOG performance status (with HR increasing from 10.47 to 50.92 for scores 1 to 3, all *p* < 0.01) and medical unfitness for intensive therapy (HR 6.45, CI: 1.99–20.88, *p* = 0.002). In the multivariate model, CD47 (HR 1.01, CI: 1.00–1.01, *p* = 0.003) and ECOG performance status (with HR increasing from 30.50 to 982.64 for scores 1 to 3, all *p* < 0.01) remained associated with survival independently from other variables, including the 2022 ELN genetic risk stratification.

## Discussion

CD47 is a transmembrane protein overexpressed in hematological malignancies and represents an innate immune checkpoint as it binds to its receptor SIRPα on macrophages inhibiting phagocytosis and favoring the immune evasion of tumor cells.

CD47-SIRPα signaling pathway is an attractive target for novel therapies in hematological malignancies, indeed blockade of this axis can increase cancer cell clearance by macrophages and augment antigen cross-presentation for T-cell priming, thus enhancing both innate and adaptive anti-cancer immune response. For this reason, several CD47-SIRPα targeting agents are under development in clinical trials [18].

The prognostic role of CD47 expression in AML varies among studies. In 2009, Majeti el al. demonstrated higher expression of CD47 on AML leukemic stem cells (LSC) than their normal counterparts and worse OS and event-free survival in AML patients with high levels of CD47 [9]. Galli et al. found that CD47 expression by immunohistochemistry (IHC) significantly correlated with BM and PB blast percentage and high CD47 expression was associated with NPM1 mutations, in contrast, CD47 expression did not significantly correlate with OS, PFS or response to therapy [10]. Recently, Marra et al. proved that CD47 expression by IHC varies according to genotype with higher expression in AML patients with favorable risk, especially those with CBFβ/MYH11 and NPM1 mutation [11]. Interestingly, Tanaka et al. demonstrated longer OS in patients with CD47 positive by IHC myeloid sarcoma, which is considered the solid variant of AML [12].

In this study CD47 expression was measured by flow cytometry on AML blasts and was compared to the residual normal lymphocytic population, which is known to express high levels of CD47 in order to avoid phagocytosis while interacting with macrophages. Only 5/35 (14.3%) patients showed higher expression of CD47 on AML blasts than in the control lymphocytic population, in fact no significant differences were found in these patients in terms of prognosis. This result may indicate that the lymphocytic population is not the ideal control for CD47 expression on AML blasts.

For this reason, it was decided to consider CD47 expression as a continuous variable and, despite the low size of the sample, it was demonstrated that increased expression of CD47 on AML blasts is related to a statistically significant reduced OS. Additionally, CD47 overexpression was associated with hyperleukocytosis and increased blast count, which are traditionally considered adverse prognostic factors [19]. However, high expression of CD47 was not related to PFS and statistical analysis did not find a cut-off value to define CD47 overexpression as predictive marker for death, relapse or response to treatment, probably due to limited sample size.

CD47 expression on AML leukemic cells was also compared to the PD-L1 expression, which is also overexpressed by AML cells to evade immune surveillance of T cells, but the two immune checkpoint inhibitors did not seem to be related in our study.

However, the CD47- SIRPα and the PD1-PD-L1 pathways are part of a more complex tumor microenvironment, which does not only include macrophages and T cells, but is also constituted by B cells, dendritic cells, stromal cells, blood vessels, and extracellular matrix. All these components interact with each other and interplay with malignant cells to actively promote tumor growth and survival. Thus, the composition of tumor microenvironment is determined by multiple factors and the same “actors” may play different roles depending on context and cancer type to define an anti-tumor microenvironment or an immune suppressive microenvironment [20]. For instance, tumor-associates macrophages (TAM), a relevant part of the tumor microenvironment in hematologic malignancies, are polarized towards form M1 (classically activated), with a pro-inflammatory phenotype resulting in tumor suppression, or M2 (alternatively activated), capable to induce angiogenesis and immune suppression leading to tumor progression, depending on the stimuli derived by cancer cells and the rest of the microenvironment [21].

AML leukemic cells are recognized to have the capacity to polarize TAM towards M2 form, which contributes to create a permissive tumor microenvironment and to promote tumor progression, acquired drug resistance and, thus, poor clinical outcome [22].

Therefore, the prognostic role of the CD47-SIRPα axis should be contextualized in the complexity of the tumor microenvironment and future studies should investigate the relation between AML leukemic cells and every component of tumor microenvironment.

In conclusion, despite the small number of patients, CD47 overexpression was associated to reduced survival in this study. However, further studies with larger sample sizes are necessary to better define the prognostic value of CD47 in patients with AML and, possibly, to identify a subgroup of patients who could derive maximum benefit from emerging CD47-SIRPα blocking therapies as a precision medicine approach.

## Data Availability

Data is provided within the manuscript.

## References

[1] Shimony S, Stahl M, Stone RM (2023) Acute myeloid leukemia: 2023 update on diagnosis, risk-stratification, and management. Am J Hematol 98(3):502–526 36594187 10.1002/ajh.26822

[2] Juliusson G, Antunovic P, Derolf A, Lehmann S, Möllgård L, Stockelberg D, Tidefelt U, Wahlin A, Höglund M (2009) Age and acute myeloid leukemia: real world data on decision to treat and outcomes from the Swedish acute leukemia registry. Blood 113(18):4179–4187 19008455 10.1182/blood-2008-07-172007

[3] Sasaki K, Ravandi F, Kadia TM, DiNardo CD, Short NJ, Borthakur G, Jabbour E, Kantarjian HM (2021) De Novo acute myeloid leukemia: A population-based study of outcome in the united States based on the surveillance, epidemiology, and end results (SEER) database, 1980 to 2017. Cancer 127(12):2049–2061 33818756 10.1002/cncr.33458 PMC11826308

[4] Russ A, Hua AB, Montfort WR, Rahman B, Riaz IB, Khalid MU, Carew JS, Nawrocki ST, Persky D, Anwer F (2018) Blocking don’t eat me signal of CD47-SIRPα in hematological malignancies, an in-depth review. Blood Rev 32(6):480–489 29709247 10.1016/j.blre.2018.04.005 PMC6186508

[5] Zhao H, Song S, Ma J, Yan Z, Xie H, Feng Y, Che S (2022) CD47 as a promising therapeutic target in oncology. Front Immunol 13:757480 36081498 10.3389/fimmu.2022.757480 PMC9446754

[6] Cao H, Wu T, Zhou X, Xie S, Sun H, Sun Y, Li Y (2023) Progress of research on PD-1/PD-L1 in leukemia. Front Immunol 14:1265299 37822924 10.3389/fimmu.2023.1265299 PMC10562551

[7] Eladl E, Tremblay-LeMay R, Rastgoo N, Musani R, Chen W, Liu A, Chang H (2020) Role of CD47 in hematological malignancies. J Hematol Oncol 13(1):96 32677994 10.1186/s13045-020-00930-1 PMC7364564

[8] Jaiswal S, Jamieson CH, Pang WW, Park CY, Chao MP, Majeti R, Traver D, van Rooijen N, Weissman IL (2009) CD47 is upregulated on Circulating hematopoietic stem cells and leukemia cells to avoid phagocytosis. Cell 138(2):271–285 19632178 10.1016/j.cell.2009.05.046 PMC2775564

[9] Majeti R, Chao MP, Alizadeh AA, Pang WW, Jaiswal S, Gibbs KD Jr, van Rooijen N, Weissman IL (2009) CD47 is an adverse prognostic factor and therapeutic antibody target on human acute myeloid leukemia stem cells. Cell 138(2):286–299 19632179 10.1016/j.cell.2009.05.045 PMC2726837

[10] Galli S, Zlobec I, Schürch C, Perren A, Ochsenbein AF, Banz Y (2015) CD47 protein expression in acute myeloid leukemia: A tissue microarray-based analysis. Leuk Res 39(7):749–756 25943033 10.1016/j.leukres.2015.04.007

[11] Marra A, Akarca AU, Martino G, Ramsay A, Ascani S, Perriello VM, O’Nions J, Wilson AJ, Gupta R, Childerhouse A, Proctor I, Rodriguez-Justo M, Pomplun S, Martelli MP, Lo Celso C, Falini B, Marafioti T (2023) CD47 expression in acute myeloid leukemia varies according to genotype. Haematologica 108(12):3491–3495 37381766 10.3324/haematol.2023.283154 PMC10690904

[12] Tanaka K, Miyoshi H, Kawamoto K, Shimasaki Y, Nakashima K, Imamoto T, Yamada K, Takeuchi M, Moritsubo M, Furuta T, Kohno K, Tamura S, Sonoki T, Ohshima K (2024) Clinicopathological analysis of CD47 and signal regulatory protein alpha expression in myeloid sarcoma patients: CD47 expression is a favourable prognostic factor. Pathology 56(1):81–91 38110323 10.1016/j.pathol.2023.10.007

[13] Chao MP, Takimoto CH, Feng DD, McKenna K, Gip P, Liu J, Volkmer JP, Weissman IL, Majeti R (2020) Therapeutic targeting of the macrophage immune checkpoint CD47 in myeloid malignancies. Front Oncol 9:1380 32038992 10.3389/fonc.2019.01380 PMC6990910

[14] Jiang C, Sun H, Jiang Z, Tian W, Cang S, Yu J (2024) Targeting the CD47/SIRPα pathway in malignancies: recent progress, difficulties and future perspectives. Front Oncol 14:1378647 39040441 10.3389/fonc.2024.1378647 PMC11261161

[15] Arber DA, Orazi A, Hasserjian RP, Borowitz MJ, Calvo KR, Kvasnicka HM, Wang SA, Bagg A, Barbui T, Branford S, Bueso-Ramos CE, Cortes JE, Dal Cin P, DiNardo CD, Dombret H, Duncavage EJ, Ebert BL, Estey EH, Facchetti F, Foucar K, Gangat N, Gianelli U, Godley LA, Gökbuget N, Gotlib J, Hellström-Lindberg E, Hobbs GS, Hoffman R, Jabbour EJ, Kiladjian JJ, Larson RA, Le Beau MM, Loh ML, Löwenberg B, Macintyre E, Malcovati L, Mullighan CG, Niemeyer C, Odenike OM, Ogawa S, Orfao A, Papaemmanuil E, Passamonti F, Porkka K, Pui CH, Radich JP, Reiter A, Rozman M, Rudelius M, Savona MR, Schiffer CA, Schmitt-Graeff A, Shimamura A, Sierra J, Stock WA, Stone RM, Tallman MS, Thiele J, Tien HF, Tzankov A, Vannucchi AM, Vyas P, Wei AH, Weinberg OK, Wierzbowska A, Cazzola M, Döhner H, Tefferi A (2022) International consensus classification of myeloid neoplasms and acute leukemias: integrating morphologic, clinical, and genomic data. Blood 140(11):1200–1228 35767897 10.1182/blood.2022015850 PMC9479031

[16] Khoury JD, Solary E, Abla O, Akkari Y, Alaggio R, Apperley JF, Bejar R, Berti E, Busque L, Chan JKC, Chen W, Chen X, Chng WJ, Choi JK, Colmenero I, Coupland SE, Cross NCP, De Jong D, Elghetany MT, Takahashi E, Emile JF, Ferry J, Fogelstrand L, Fontenay M, Germing U, Gujral S, Haferlach T, Harrison C, Hodge JC, Hu S, Jansen JH, Kanagal-Shamanna R, Kantarjian HM, Kratz CP, Li XQ, Lim MS, Loeb K, Loghavi S, Marcogliese A, Meshinchi S, Michaels P, Naresh KN, Natkunam Y, Nejati R, Ott G, Padron E, Patel KP, Patkar N, Picarsic J, Platzbecker U, Roberts I, Schuh A, Sewell W, Siebert R, Tembhare P, Tyner J, Verstovsek S, Wang W, Wood B, Xiao W, Yeung C, Hochhaus A (2022) The 5th edition of the World Health Organization Classification of Haematolymphoid Tumours: Myeloid and Histiocytic/Dendritic Neoplasms. Leukemia 36(7):1703–1719 10.1038/s41375-022-01613-1 PMC9252913 35732831

[17] Döhner H, Wei AH, Appelbaum FR, Craddock C, DiNardo CD, Dombret H, Ebert BL, Fenaux P, Godley LA, Hasserjian RP, Larson RA, Levine RL, Miyazaki Y, Niederwieser D, Ossenkoppele G, Röllig C, Sierra J, Stein EM, Tallman MS, Tien HF, Wang J, Wierzbowska A, Löwenberg B (2022) Diagnosis and management of AML in adults: 2022 recommendations from an international expert panel on behalf of the ELN. Blood 140(12):1345–1377 35797463 10.1182/blood.2022016867

[18] Jia X, Yan B, Tian X, Liu Q, Jin J, Shi J, Hou Y (2021) CD47/SIRPα pathway mediates cancer immune escape and immunotherapy. Int J Biol Sci 17(13):3281–3287 34512146 10.7150/ijbs.60782 PMC8416724

[19] Dutcher JP, Schiffer CA, Wiernik PH (1987) Hyperleukocytosis in adult acute nonlymphocytic leukemia: impact on remission rate and duration, and survival. J Clin Oncol 5(9):1364–1372 3625254 10.1200/JCO.1987.5.9.1364

[20] Anderson NM, Simon MC (2020) The tumor microenvironment. Curr Biol 30(16):R921–R925 32810447 10.1016/j.cub.2020.06.081 PMC8194051

[21] Cencini E, Fabbri A, Sicuranza A, Gozzetti A, Bocchia M (2021) The role of Tumor-Associated macrophages in hematologic malignancies. Cancers (Basel) 13(14):3597 34298810 10.3390/cancers13143597 PMC8304632

[22] Al-Matary YS, Botezatu L, Opalka B, Hönes JM, Lams RF, Thivakaran A, Schütte J, Köster R, Lennartz K, Schroeder T, Haas R, Dührsen U, Khandanpour C (2016) Acute myeloid leukemia cells polarize macrophages towards a leukemia supporting state in a growth factor independence 1 dependent manner. Haematologica 101(10):1216–1227 27390361 10.3324/haematol.2016.143180 PMC5046651

